# Supplementary material for: Why Do Children in Slums Suffer from Anemia, Iron, Zinc, and Vitamin A Deficiency? Results from a Birth Cohort Study in Dhaka
Source: Nutrients. 2019 Dec 11;11(12):3025. doi: 10.3390/nu11123025 (PMC6949995; doi:10.3390/nu11123025)
Supplement: Supplementary file 1 [file nutrients-11-03025-s001.pdf]

Supplementary Materials-1

S1. Supplementary Tables

Table S1: Availability of different variables across different time points

Variables	7 months	15 months	24 months	60 months
SES, WAMI	+	+	+	+
Maternal age, education, age at marriage	+	+	+	+
Household income, asset	+	+	+	+
WASH behavior	+	+	+	+
Morbidity and antibiotic use	+	+	+	-
Stool microbiology	+	+	+	-
EED: MPO, NEO, AAT	+	+	+	-
Anthropometry	+	+	+	+
Zn, retinol, ferritin, Hb, sTFR, CRP, AGP	+	+	+	+
Child feeding: EBF, complementary feeding	+	+	+	+

Table S2 Bivariate analysis for anemia and other micronutrient deficiencies

	Anemia	Zinc deficiency	Vitamin A deficiency	Iron deficiency
	**OR (95% CI), p-value**	**OR (95% CI), p-value**	**OR (95% CI), p-value**	**OR (95% CI), p-value**
Inflammation group				
Non-inflammed	Reference	Reference	Reference	Reference
Incubation	2.77 (1.09, 7.04), 0.032	1	1.37 (0.46, 4.08), 0.577	1.15 (0.45, 2.91), 0.770
Early convalescence	2.53 (1.56, 4.10), <0.001	1.04 (0.48, 2.24), 0.923	0.99 (0.55, 1.75), 0.962	0.96 (0.59, 1.58), 0.877
Late convalescence	2.16 (1.46, 3.17), <0.001	1.36 (0.73, 2.55), 0.335	1.14 (0.74, 1.77), 0.557	1.07 (0.70, 1.62), 0.764
Age in months	0.94 (0.93, 0.96), <0.001	0.95 (0.93, 0.98), <0.001	0.96 (0.95, 0.97), <0.001	0.97 (0.49, 0.91), 0.011
Female	0.63 (0.44, 0.89), 0.010	0.79 (0.50, 1.25), 0.313	0.85 (0.58, 1.26), 0.422	0.67 (0.44, 0.89), 0.010
Birth weight	0.64 (0.40, 1.03), 0.066	0.93 (0.55, 1.59), 0.792	0.73 (0.47, 1.13), 0.160	0.62 (0.41, 0.94), 0.024
Underweight (WAZ <-2) at birth	1.21 (0.78, 1.87), 0.388	1.02 (0.61, 1.72), 0.936	1.13 (0.73, 1.75), 0.586	1.67 (1.16, 1.41), 0.006
Stunting (LAZ <-2) at birth	1.19 (0.71, 2.02), 0.503	0.89 (0.49, 1.61), 0.696	0.96 (0.59, 1.55), 0.866	1.09 (0.72, 1.67), 0.663
Wasting (WLZ <-2) at birth	1.39 (0.93, 2.09), 0.108	1.56 (0.95, 2.55), 0.080	0.92 (0.56, 1.52), 0.753	1.37 (0.91, 2.08), 0.131
Days of exclusive breast feeding	1.00 (0.99, 1.00), 0.442	0.99 (0.99, 1.00), 0.437	0.99 (0.99, 1.00), 0.315	0.99 (0.99, 1.00), 0.785
Food insecurity access				
Food secure	Reference	Reference	Reference	Reference
Mild food insecurity	1.09 (0.47, 2.57), 0.828	0.84 (0.32, 2.16), 0.712	1.22 (0.5, 2.69), 0.626	0.87 (0.40, 1.86), 0.713
Moderate food insecurity	0.75 (0.44, 1.28), 0.291	0.82 (0.40, 1.67), 0.591	0.91 (0.48, 1.73), 0.775	1.18 (0.77, 1.81), 0.446
Severe food insecurity	1.39 (0.77, 2.50), 0.274	0.59 (0.22, 1.57), 0.296	2.62 (1.35, 5.07), 0.004	1.33 (0.74, 2.38), 0.347
Monthly income	0.99 (0.99, 1.00), 0.138	1.00 (0.99, 1.00), 0.105	0.99 (0.99, 0.99), 0.016	0.99 (0.99, 1.00), 0.776
Asset index				

Poorest	Reference	Reference	Reference	Reference
Poor	0.68 (0.39, 1.17), 0.162	1.34 (0.66, 2.74), 0.420	0.75 (0.41, 1.37), 0.354	0.87 (0.52, 1.47), 0.609
Middle	0.81 (45, 1.48), 0.496	0.97 (0.41, 2.32), 0.950	1.11 (0.63, 1.94), 0.726	1.06 (0.64, 1.77), 0.818
Wealthier	0.71 (0.41, 1.23), 0.217	1.14 (0.53, 2.45), 0.743	0.58 (0.33, 1.02), 0.057	1.12 (0.69, 1.79), 0.642
Wealthiest	0.64 (0.35, 1.19), 0.158	1.71 (0.85, 2.45), 0.131	0.29 (0.15, 0.57), <0.001	1.01 (0.58, 1.75), 975
Maternal education				
No	Reference	Reference	Reference	Reference
Some primary	0.96 (0.58, 1.59), 0.865	0.73 (0.38, 1.39), 0.341	0.64 (0.34, 1.18), 0.152	1.11 (0.70, 1.76), 0.651
Primary complete	0.81 (0.45, 1.48), 0.496	0.77 (0.38, 1.55), 0.461	1.09 (0.59, 2.05), 0.766	1.40 (0.85, 2.32), 0.187
Some secondary	0.69 (0.42, 1.14), 0.151	0.64 (0.34, 1.21), 0.169	0.78 (0.44, 1.41), 0.420	0.82 (0.59, 1.42), 0.696
Secondary complete or higher	0.59 (0.24, 1.44), 0.247	0.34 (0.09, 1.32), 0.120	0.98 (0.39, 2.50), 0.973	2.05 (0.98, 4.29), 0.055
Treat drinking water	0.93 (0.64, 1.34), 0.698	1.31 (0.82, 2.12), 0.260	0.45 (0.31, 0.67), <0.001	1.13 (0.81, 1.58), 0.470
Improved toilet	0.95 (0.62, 1.45), 0.813	0.66 (0.41, 1.08), 0.100	1.56 (0.97, 2.49), 0.064	1.02 (0.72, 1.46), 0.905
Hand washing after help the child defecate	1.81 (1.18, 2.77), 0.007	1.78 (0.96, 3.17), 0.052	0.82 (0.53, 1.26), 0.364	1.26 (0.87, 1.82), 0.219
Hand washing before food preparation	1.31 (0.87, 1.99), 0.198	1.11 (0.64, 1.94), 0.703	0.99 (0.63, 1.58), 1.00	1.34 (0.91, 1.96), 0.137
Hand washing after using toilet	1.59 (1.02, 2.51), 0.043	1.75 (0.99, 3.06), 0.051	0.69 (0.43, 1.11), 0.124	1.26 (0.87, 1.85), 0.212
Age of mother in year	0.98 (0.93, 1.00), 0.064	0.99 (0.95, 1.04), 0.797	0.98 (0.94, 1.02), 0.317	0.96 (0.93, 0.99), 0.040
Anemia		1.56 (1.01, 2.42), 0.047	2.89 (2.04, 4.12), <0.001	3.79 (2.72, 5.29), <0.001
Zinc deficiency	1.59 (1.04, 2.45), 0.33		1.70 (1.06, 2.72), 0.027	0.94 (0.59, 1.48), 0.790
Iron deficiency	3.59 (2.59, 4.99), <0.001	0.96 (0.69, 1.52), 0.849	1.39 (0.99, 1.95), 0.050	
Vitamin A deficiency	2.86 (2.02, 4.03), <0.001	1.61 (0.99, 2.64), 0.057		1.40 (1.00, 1.97), 0.049

Table S3 Fit statistics of the final models derived using LCGM for Hemoglobin, Retinol and Zinc

Trajectory group	n	Average posterior probability	Odds of correct classification	Estimated group probability	Proportion of group assignment
Hemoglobin (g/dl)					
Group 1 (Decreasing)	39	0.88	7.5	0.28	0.25
Group 2 (Increasing)	116	0.93	12.8	0.72	0.75
Ferritin (ng/ml)					
Group 1 (Lower)	132	0.99	115.5	0.86	0.86
Group 2 (Higher)	21	0.93	13.9	0.14	0.14
Retinol (µg/dl)					
Group 1 (Lower)	138	0.98	39.0	0.88	0.90
Group 2 (Higher)	16	0.90	9.1	0.12	0.10
Zinc (mmol/L)					
All children	155	1.0	-	1.0	1.0

Supplementary Materials-2

2.1 Measurement of plasma micronutrient status

Plasma zinc was measured using flame atomic absorption spectrophotometry (Shimadzu AA-6501S, Kyoto, Japan). A four point calibration curve was prepared in every lot from the commercial zinc standard solution (Cica-Reagent, Kanto Chemical Co. INC), in concentrations of 0.1, 0.2, 0.3 and 0.4 mg/L. Diluted plasma was then aspirated into the AAS. Results were calculated from a standard curve [1].

For retinol assays, plasma was deproteinized with methanol containing retinyl acetate (Sigma Chemical Co., St Louis, MO, USA) as an internal standard and retinol was extracted twice with hexane. The hexane layer was pooled and evaporated under nitrogen gas. Then residue was re-dissolved in the mobile phase (95% methanol) and injected into HPLC (SPD-10A vp uv-visible detector, LC-10AT vp solvent delivery system and Chromatopac C-R8A integrator, Shimadzu, LC, Kyoto, Japan). Retinol was separated by reverse phase HPLC using C18 column (Discovery C18, 25cmX4mm, 5µm, Cat# 504971) and detected at 325nm. A pooled human plasma sample was calibrated against standard reference material (fat-soluble vitamins, carotenoids and cholesterol in human serum, 968c; National Institute of Standards and Technology, Gaithersburg, MD, USA). Three aliquots of the plasma pool were analyzed with each set of samples, and the retinol concentration was calculated based on the known concentration of retinol in the plasma pool [2].

Ferritin, CRP and AGP were determined by Immunoturbidimetric assay using commercial kits from Roche diagnostics on Roche automated clinical chemistry analyzer Hitachi -902 (Boehringer Mannheim, Germany). A HemoCue 201 machine was used to measure hemoglobin concentration [3].

2.2 Latent class growth modeling (LCGM) and multiple linear regression models

We used latent class growth modeling (LCGM), also called group-based trajectory modeling, to identify distinct clusters or classes of children following similar trajectories with regard to the pattern of hemoglobin, ferritin, retinol and zinc during the age of 7 to 24 months. LCGM is a semi-parametric, finite mixture modeling technique which analyzes longitudinal data using maximum likelihood to identify meaningful and distinct groups of individuals who follow similar progression over time for a given variable. LCGM relaxes the assumption that all individuals are drawn from a single population and allows for differences in growth parameters across unobserved subpopulations. However, it assumes that the intercept and slope are fixed for all individuals within each distinct group [4-5].

We built separate trajectory models for hemoglobin, ferritin, retinol and zinc. LCGM requires at least three measurement time-points for each case to generate reliable parameter estimates of trajectories [6]. Therefore, the analyses were restricted to the children for whom data on the outcomes were available at 7, 15 and 24 months (three time-points). In addition, for ferritin, three very large and unusual values were dropped from the dataset. The sample sizes for LCGM were reduced to 155 for hemoglobin, 153 for ferritin, 154 for retinol and 155 for zinc.

We built and compared several models for each outcome with 1 to 4 trajectories. A censored normal distribution approach was used for modeling all three outcomes. To identify the optimal number of trajectories, we fit models of increasing complexity starting from a single trajectory and finalized the model that best fit the data. In identifying the distinct trajectories of the outcomes, linear and quadratic functions of time (age in months) were examined. Non-significant quadratic terms were removed from a model for a given trajectory. Models generating

trajectories with insufficient cluster size (less than 5% of the study population) were not considered.

We selected the final models with optimal number and shape of trajectories based on Bayesian information criteria (BIC), log Bayes factor, the statistical significance of quadratic terms, whether 95% confidence intervals of trajectories overlapped, and the percentage of the population in each trajectory group. The smallest absolute value of the BIC indicated the best fit. A value greater than 6 for the estimated log Bayes factor, which is equal to two times the difference in the BIC values calculated by subtracting the BIC of the simpler model from that of the more complex model, was interpreted as strong evidence for the more complex model [7]. After selecting the final model, we calculated the posterior probabilities for each individual of belonging to each of the trajectory groups, and individuals were assigned to a trajectory group based on the maximum-probability assignment rule [8].

We used the following criteria to assess the goodness of fit of the final models: whether the average posterior probability of assignment was greater than 0.8 for each of the subgroups, whether the odds of correct classification was greater than 5, and whether the estimated/modeled group probabilities were in good agreement with the proportions of group assignments [27]. We reported the findings of the LCGM following *The GRoLTS-Checklist: Guidelines for Reporting on Latent Trajectory Studies* [9].

In subsequent analyses, multiple linear regression models with robust standard errors were fitted to examine the association of levels of hemoglobin, ferritin, retinol and zinc at the age of 60 months with the trajectories identified through LCGM. As zinc was found to have a single trajectory (described in detail in the results section), level of zinc at 24 months was used instead

of any trajectory to assess the predictive association with level of zinc at 60 months. We considered several covariates collected at the age of 60 months in building these models which include sum of energy in Kcal for the day of food recall, sum of protein in grams for the day of food recall, sum of fat in grams for the day of food recall, sum of carbohydrates in grams for the day of food recall, sum of iron in mg for the day of food recall, sum of Vitamin A in ug for the day of food recall, sum of zinc in mg for the day of food recall, phytate to iron ratio of the diet, phytate to zinc ratio of the diet, energy in Kcal from carbohydrates (minus fiber) as percent of total energy, energy in Kcal from protein as percent of total energy, energy in Kcal from fat as percent of total energy, socioeconomic and WASH variables including WAMI score, and child sex. However, the final regression models included only iron intake, the percentage of energy from protein, and WAMI score for hemoglobin, level of zinc at 24 months, total energy intake, protein intake and WAMI score for ferritin, vitamin A intake and WAMI score for retinol, and zinc intake and WAMI score for zinc. Due to missing values for the outcome variables, the sample sizes for the linear models were reduced to 142 for hemoglobin, 138 for ferritin and retinol and 140 for zinc.

The statistical analyses related to LCGM were performed using the "traj plugin" in Stata (StataCorp, College Station, Texas 77845 USA, version 14.1) [10], a Stata equivalent of the widely used "proc traj" in SAS [28]. Outputs of LCGM models were plotted using "traj" in Stata and the R packages "lcmm" and "ggplot2" in R (version 3.5.1). All other statistical analyses were performed with Stata/PC (StataCorp, College Station, Texas 77845 USA, version 14.1).

Reference

1. AOAC Official Methods of Analysis (1990) 15th ed. 2nd Supplement (1991) p: 81

2. Driskell WJ, Neese JW, Bryant CC, Bashor MM. Measurement of vitamin A in human serum by high-performance liquid chromatography, J Chromatogr. 1982 Sep 10; 231(2):439-44.

3. Caulfield LE, Bose A, Chandyo RK, Nesamvuni C, de Moraes ML, Turab A et al. Infant Feeding Practices, Dietary Adequacy, and Micronutrient Status Measures in the MAL-ED Study. Clin Infect Dis. 2014 Nov; 59: S248–S254.

4. Nagin DS. Analyzing developmental trajectories: a semiparametric, group-based approach. Psychol Methods. 1999;4(2):139.

5. Nagin DS, Tremblay RE. Analyzing developmental trajectories of distinct but related behaviors: a group-based method. Psychol Methods. 2001;6(1):18.

6. Nagin DS, NAGIN D. Group-based modeling of development: Harvard University Press; 2005.

7. Jones BL, Nagin DS, Roeder K. A SAS procedure based on mixture models for estimating developmental trajectories. Sociological methods & research. 2001;29(3):374-93.

8. Andruff H, Carraro N, Thompson A, Gaudreau P, Louvet B. Latent class growth modelling: a tutorial. Tutorials in Quantitative Methods for Psychology. 2009;5(1):11-24.

9. Van De Schoot R, Sijbrandij M, Winter SD, Depaoli S, Vermunt JK. The GRoLTS-checklist: guidelines for reporting on latent trajectory studies. Structural Equation Modeling: A Multidisciplinary Journal. 2017;24(3):451-67.

10. Jones BL, Nagin DS. A note on a Stata plugin for estimating group-based trajectory models. Sociological Methods & Research. 2013;42(4):608-13.

Checklist

STROBE Statement—Checklist of items that should be included in reports of ***cohort studies***

	Item No	Recommendation	Page No
**Title and abstract**	1	(*a*) Indicate the study's design with a commonly used term in the title or the abstract	1
		(*b*) Provide in the abstract an informative and balanced summary of what was done and what was found	3
**Introduction**			
Background/rationale	2	Explain the scientific background and rationale for the investigation being reported	5,6
Objectives	3	State specific objectives, including any prespecified hypotheses	6
**Methods**			
Study design	4	Present key elements of study design early in the paper	6,7
Setting	5	Describe the setting, locations, and relevant dates, including periods of recruitment, exposure, follow-up, and data collection	5,6
Participants	6	(*a*) Give the eligibility criteria, and the sources and methods of selection of participants. Describe methods of follow-up	7,8,9
		(*b*) For matched studies, give matching criteria and number of exposed and unexposed	
Variables	7	Clearly define all outcomes, exposures, predictors, potential confounders, and effect modifiers. Give diagnostic criteria, if applicable	7,8,9
Data sources/ measurement	8*	For each variable of interest, give sources of data and details of methods of assessment (measurement). Describe comparability of assessment methods if there is more than one group	7,8,9
Bias	9	Describe any efforts to address potential sources of bias	9,10,11,12
Study size	10	Explain how the study size was arrived at	12
Quantitative variables	11	Explain how quantitative variables were handled in the analyses. If applicable, describe which groupings were chosen and why	10-12
Statistical methods	12	(*a*) Describe all statistical methods, including those used to control for confounding	9-11
		(*b*) Describe any methods used to examine subgroups and interactions	11
		(*c*) Explain how missing data were addressed	
		(*d*) If applicable, explain how loss to follow-up was addressed	
		(*e*) Describe any sensitivity analyses	11, S1.2
**Results**			
Participants	13*	(a) Report numbers of individuals at each stage of study—eg numbers potentially eligible, examined for eligibility, confirmed eligible, included in the study, completing follow-up, and analysed	13
		(b) Give reasons for non-participation at each stage	11

		(c) Consider use of a flow diagram	
Descriptive data	14*	(a) Give characteristics of study participants (eg demographic, clinical, social) and information on exposures and potential confounders<br>(b) Indicate number of participants with missing data for each variable of interest<br>(c) Summarise follow-up time (eg, average and total amount)	Table 1<br><br><br><br>12
Outcome data	15*	Report numbers of outcome events or summary measures over time	12-13
Main results	16	(*a*) Give unadjusted estimates and, if applicable, confounder-adjusted estimates and their precision (eg, 95% confidence interval). Make clear which confounders were adjusted for and why they were included<br>(*b*) Report category boundaries when continuous variables were categorized<br>(*c*) If relevant, consider translating estimates of relative risk into absolute risk for a meaningful time period	12-15<br><br><br>14-16<br>14-17
Other analyses	17	Report other analyses done—eg analyses of subgroups and interactions, and sensitivity analyses	14-17, supple
**Discussion**			
Key results	18	Summarise key results with reference to study objectives	17-20
Limitations	19	Discuss limitations of the study, taking into account sources of potential bias or imprecision. Discuss both direction and magnitude of any potential bias	20
Interpretation	20	Give a cautious overall interpretation of results considering objectives, limitations, multiplicity of analyses, results from similar studies, and other relevant evidence	17-20
Generalisability	21	Discuss the generalisability (external validity) of the study results	17-20
**Other information**			
Funding	22	Give the source of funding and the role of the funders for the present study and, if applicable, for the original study on which the present article is based	21

*Give information separately for exposed and unexposed groups.

Note: An Explanation and Elaboration article discusses each checklist item and gives methodological background and published examples of transparent reporting. The STROBE checklist is best used in conjunction with this article (freely available on the Web sites of PLoS Medicine at http://www.plosmedicine.org/, Annals of Internal Medicine at http://www.annals.org/, and Epidemiology at http://www.epidem.com/). Information on the STROBE Initiative is available at http://www.strobe-statement.org.
